# The effect of water storage on nanoindentation creep of various CAD-CAM composite blocks

**DOI:** 10.1186/s12903-023-03145-1

**Published:** 2023-08-07

**Authors:** Rasha A. Alamoush, Nesreen A. Salim, Alaaeldin Elraggal, Julian D. Satterthwaite, Nikolaos Silikas

**Affiliations:** 1 Department of Fixed and Removable Prosthodontics, School of Dentistry, the University of Jordan, Amman, 11942 Jordan; 2 Operative Dentistry, Conservative Dentistry Department, Faculty of Dentistry, Alexandria University, Alexandria, Egypt; 3 Division Dentistry, School of Medical Sciences, the University of Manchester, Manchester, UK

**Keywords:** CAD-CAM composite blocks, Polymer-infiltrated ceramic network, Nanoindentation creep, Polyetheretherketone

## Abstract

**Background:**

To study the effect of water storage (3 months) on the creep deformation of various CAD-CAM composite structures at the nanoscale and compare it to that at the macroscale.

**Methods:**

Seven CAD-CAM blocks were investigated: five resin-composite blocks (RCB), one polymer-infiltrated ceramic network (PICN) block, and one ceramic-filled polyetheretherketone (PEEK) block. Specimens of each material (n = 6) were separated into two groups (n = 3) according to their storage conditions (24 h dry storage at 23**˚C** and 3 months storage in 37**˚C** distilled water). Nano-indentation creep measurements were undertaken (creep depth measured in µm) using a nanoindenter (Nanovea) equipped with Berkovich three-sided pyramidal diamond tip. The machine was set for the chosen parameters: a load of 20 gf, a pause of 20 s, and the material type. Thirty indentations on 3 samples were made for each material for each test. Data were analysed using two-way ANOVA followed by one-way ANOVA and Bonferroni post hoc tests and independent t-test (< 0.05) for comparisons between the materials.

**Results:**

The nanoindentation creep depth after 24 h storage ranged from 0.09 to 0.33 μm and increased after 3 months storage in distilled water to between 0.28 and 3.46 μm. There was a statistically significant difference in nanoindentation creep behaviour between the two storage conditions for each investigated material (independent t-test) and between all materials (Bonferroni post hoc). There was a non-significant negative correlation between nanoindentation creep (µm) and filler weight% at 24 h dry storage but a significant correlation at 3 months of water storage. A further non-significant positive correlation between nanoindentation creep (µm) and bulk compressive creep (%) was found.

**Conclusion:**

The PICN material showed superior dimensional stability in terms of nanoindentation creep depth in both storage conditions. Other composite blocks showed comparable performance at 24 h dry condition, but an increased nanoindentation creep upon water storage.

## Background

Advances in adhesive dentistry techniques and technological developments using computer-aided design (CAD) and computer-aided manufacturing (CAM) systems make it possible to produce improved materials for indirect aesthetic restorations [1, 2]. CAD-CAM materials for aesthetic restorations are currently available as glass-ceramics/ceramics and resin-composites [2–4]. CAD-CAM resin composite materials are composed of a heavily filled polymer matrix polymerised under high temperature and/or pressure [5–7]. CAD-CAM composites can be classified based on their microstructure into resins with dispersed fillers (resin composite blocks: RCB) and polymer infiltrated ceramic networks [PICN] [7]. Composite with dispersed inorganic filler particles are formed using simple mixing of filler and the polymeric matrix [1, 8]. CAD-CAM resin composites blocks have a high degree of conversion [9] due to polymerisation under high pressure and high temperature [10] resulting in higher composite homogeneity with fewer flaws and pores compared to conventional indirect composites [10, 11], which also allows incorporation of higher filler content [12]. They have shown improved mechanical properties [13] such as wear resistance [14], flexural strength [15], fracture toughness, and fracture strength [16] compared to conventional indirect resin composites. PICN materials are formed as a porous pre-sintered ceramic network conditioned by a coupling agent, that is then infiltrated with a polymer [16]; PICN thus presents a three-dimensional skeleton with improved resistance to breakdown [17, 18]. Polyetheretherketone (PEEK) has also been widely used as CAD-CAM material in both fixed and removable prosthodontics fields [19].

Creep can be defined as the strain generated within a material in response to a load application [20]. Creep behaviour can be investigated using several methods such as the indentation method [21], 3-point bending [22], and compression of a cylindrical specimen [8]. Creep behaviour at the nano and macroscale has been studied extensively for conventional resin composites [23–25], however, few studies have investigated the creep behaviour of CAD-CAM composite blocks. There are four methods of nanoindentation creep measurement as described by Lucas and Oliver: indentation load relaxation method, constant rate of loading method, constant load indentation method, and impression creep method. The constant load indentation method, which records the change of depth with time, is the most commonly used method [26].

Nanoindentation was used to measure creep depth in this research as a well-documented method to investigate the micro-filled and nano-filled composites properties [27]. However, it is very sensitive to thermal changes and mechanical vibration and acoustic noise and this is considered a limitation [28].

This study aimed to assess the nanoindentation creep of different CAD-CAM blocks. The null hypotheses for the investigated materials were that (1) there is no difference in nanoindentation creep depth between the materials (2) there is no effect of water storage (3 months) on nanoindentation creep depth of CAD-CAM composite materials, (3) the nanoindentation creep will not be affected by their composition (filler weight%). (4) There is no correlation between nanoindentation creep depth and bulk compressive creep.

## Materials and methods

Seven CAD-CAM materials were investigated: five resin-composite blocks (RCB), one polymer-infiltrated ceramic network (PICN) block, and one ceramic-filled polyetheretherketone (PEEK) block (Table 1).

**Table 1:** The manufacturers’ compositional information and experimentally determined filler weight% of the materials investigated [29]

Material *(Code)*		Manufacturer	Composition by weight represented by the manufacturers		Filler weight%
			Polymer	Filler	
Polymer- infiltrated ceramic network (PICN)	Vita Enamic (EN)	Vita Zahnfabrik, Germany	14% UDMA, TEGDMA	86% fine structure feldspar ceramic	85.1(0.1)
Resin-composite blocks (RCB)	Grandio Blocs (GR)	VOCO GmbH, Germany	14% UDMA, DMA	86% nanohybrid fillers	84.6(0.01)
	Lava™ Ultimate (LU)	3 M™ESPE™, USA	20% Bis-GMA, UDMA, Bis-EMA,TEGDMA	80% silica and zirconia nano particles	74.8(0.1)
	BRILLIANT Crios (BC)	COLTENE, Switzerland	Cross-linked methacrylates (Bis-GMA, Bis-EMA, TEGDMA)	70% of glass and amorphous silica	70.1(0.05)
	Cerasmart (CS)	GC dental products, Europe	Bis-MEPP, UDMA, DMA	71% silica and barium glass nanoparticles	66.1(0.2)
	Block HC (HC)	Shofu, Japan	UDMA, TEGDMA	61% silica powder, microfumed silica, and zirconium silicate	63(0.02)
Ceramic-filled PEEK	Dentokeep (DK)	NT-Trading, Germany	80% PEEK	20% TiO_2_	27.5(0.06)

### Sample preparation

Specimens of each CAD-CAM block were sectioned using a diamond blade (MK 303; MK Diamond, CA, USA) mounted on a saw (Isomet 1000 Precision Saw; Buehler Co, IL, USA) under constant water irrigation. Specimens were wet ground and polished with a series of silicon carbide paper (SiC) (P320, P500, P1200, P2400, and P4000 grit (Buehler Co, IL, USA)) under water cooling and then polished with 0.25 μm diamond suspension (Meta Di Supreme, Buehler Co, IL, USA) using a lapping machine (MetaServ 250, Buehler Co, IL, USA). Sample size and power calculations were excuted according to mean differences and standard deviations of the initial obtained data at a confidence interval of 95%. A sample size of 3 samples was found to be sufficient with significance level of 0.05. Each sample received 10 indentation measurements, 500 μm apart, to make up a total of 30 measurments per subgroup. Therefore, a total of 42 samples were prepared; six specimens of each material. The samples were then divided into two groups (n = 3) according to their storage conditions (24 h dry storage at 23˚C versus 3 months storage in 37˚C distilled water).

### 1 Nanoindentation creep measurement

**Fig. 1:**
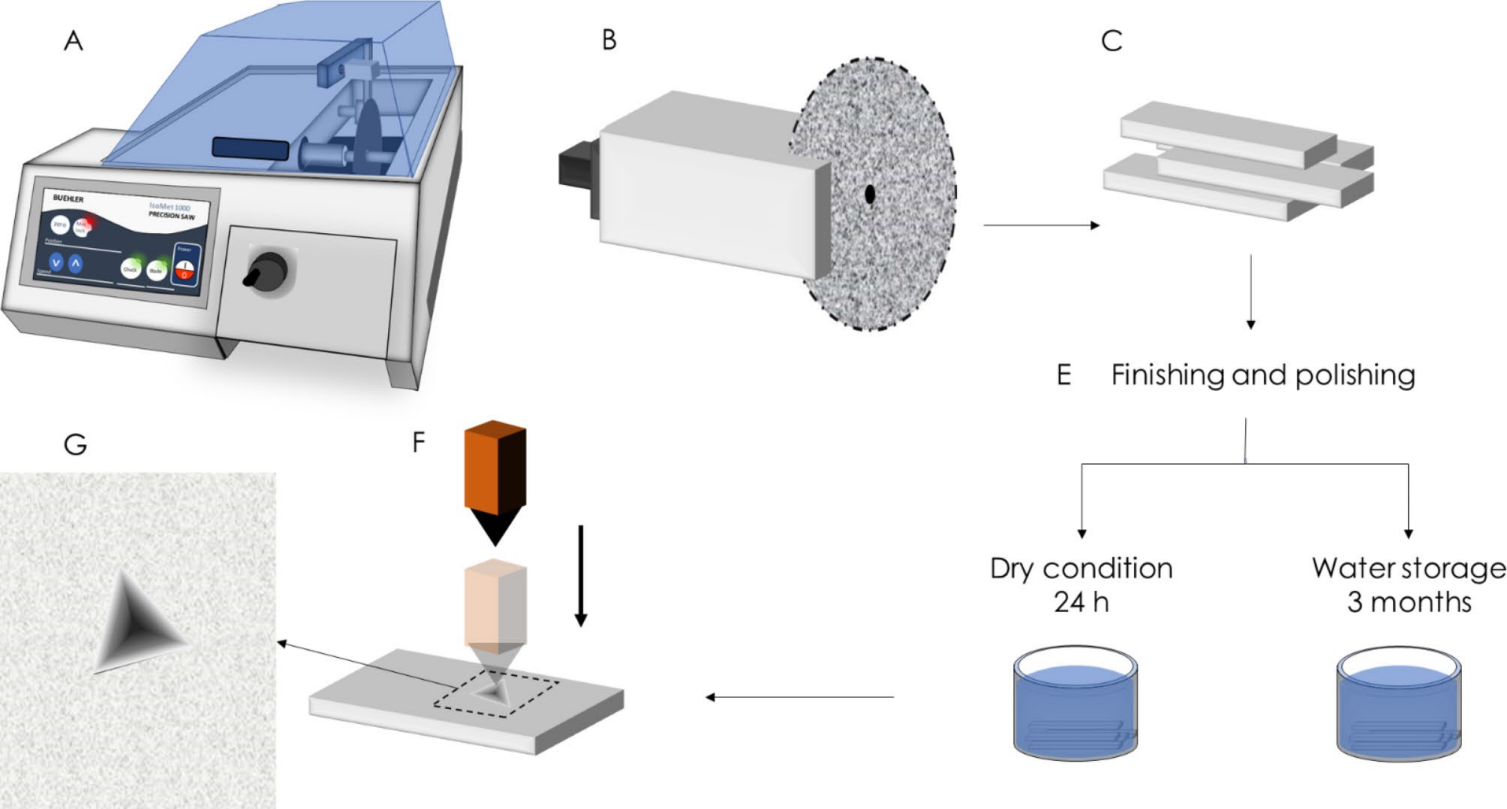
Schematic diagram showing the steps of specimens preparation (**A**-**E**) and nanoindentation testing (**F** and **G**)

Thirty indentations on 3 samples were made for each material for each test. Measurements were obtained using a nanoindenter (M3 Nanovea; Nanovea Co, CA, USA) equipped with a Berkovich three-sided pyramidal diamond tip with an indenter cone angle of 130.54° and elastic modulus of 1140 GPa. Calibration indents were made on a fused silica sample with an elastic modulus of 71.3 GPa and hardness of 8.9 GPa (Fig. 1). The machine was set for the chosen parameters: a load of 20 gf (equivalent to 200 mN), a dwell time of 20 s [21], and material type, which set the Poisson’s ratio. The nanoindentation creep was measured as displacement in µm while the sample was loaded gradually by the nanoindenter until the pre-set maximum load reached and then held for a period of time (holding time), this cause the sample to be displaced during loading and recover after unloading. the The nanoindentation creep was measured as the force was held constant (20 gf) then the penetration depth was calculated by the machine during the creep phase [30–32]. All nanoindentation measurements after storage were done at room temperature.

### Data analysis

Data were analysed using statistical software (GraphPad Prism version 8.4.3) and found to be normally distributed as verified by Shapiro-Wilk’s test. The effect of material type and water storage on nanoindentation creep was analysed using two-way analysis of variance (ANOVA) followed by one-way ANOVA and Bonferroni post hoc tests for multiple comparisons between the materials for each group. Independent sample t-test was used for the difference between the two storage groups for each individual material. Pearson correlation was used to assess filler weight and nanoindentation creep and also the correlation of nanoindentation and bulk compressive creep (tested in previous study [8]) after 24 h dry storage and 3 months water storage. All tests were conducted at a significance level of α = 0.05.

## Results

**Fig. 2:**
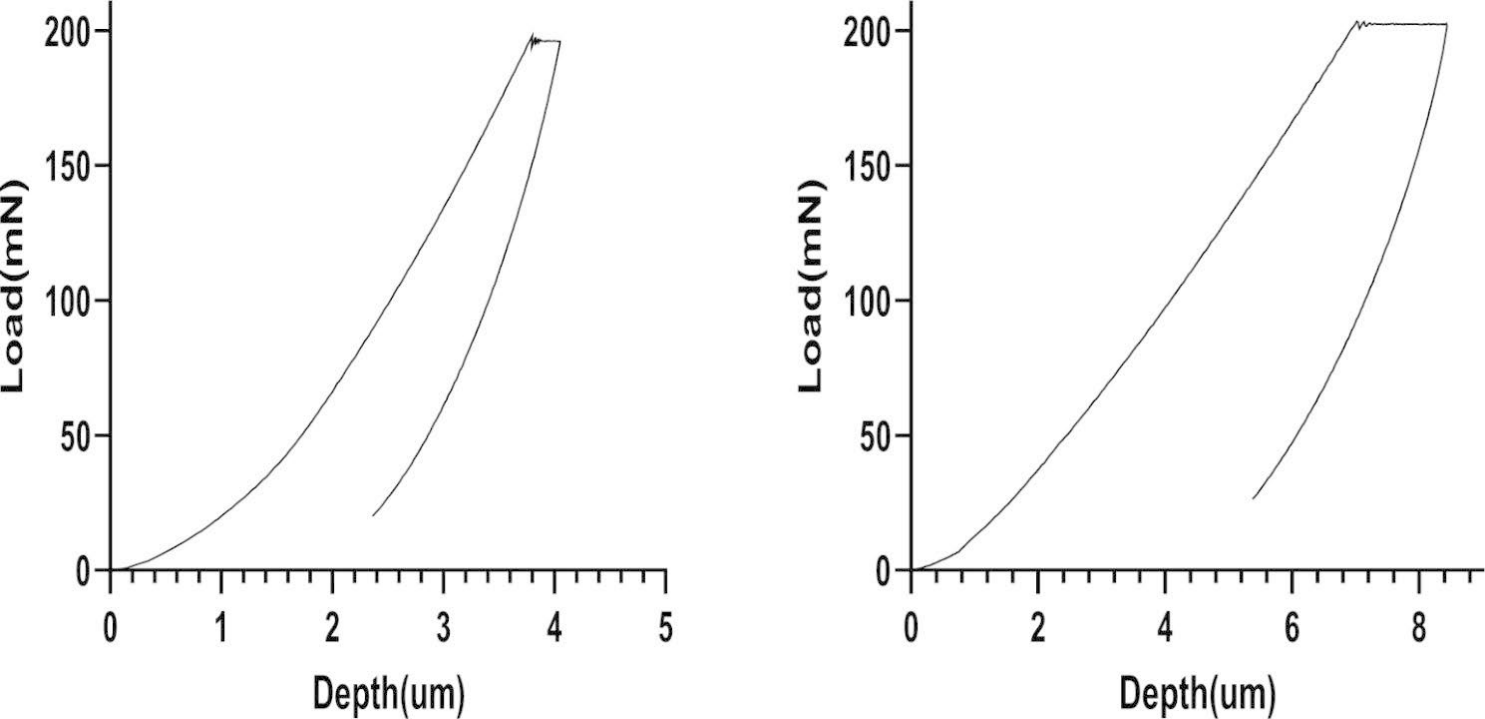
The force-displacement curve generated from nanoindentation machine at 24 h and after 3 months water storage

**Fig. 3:**
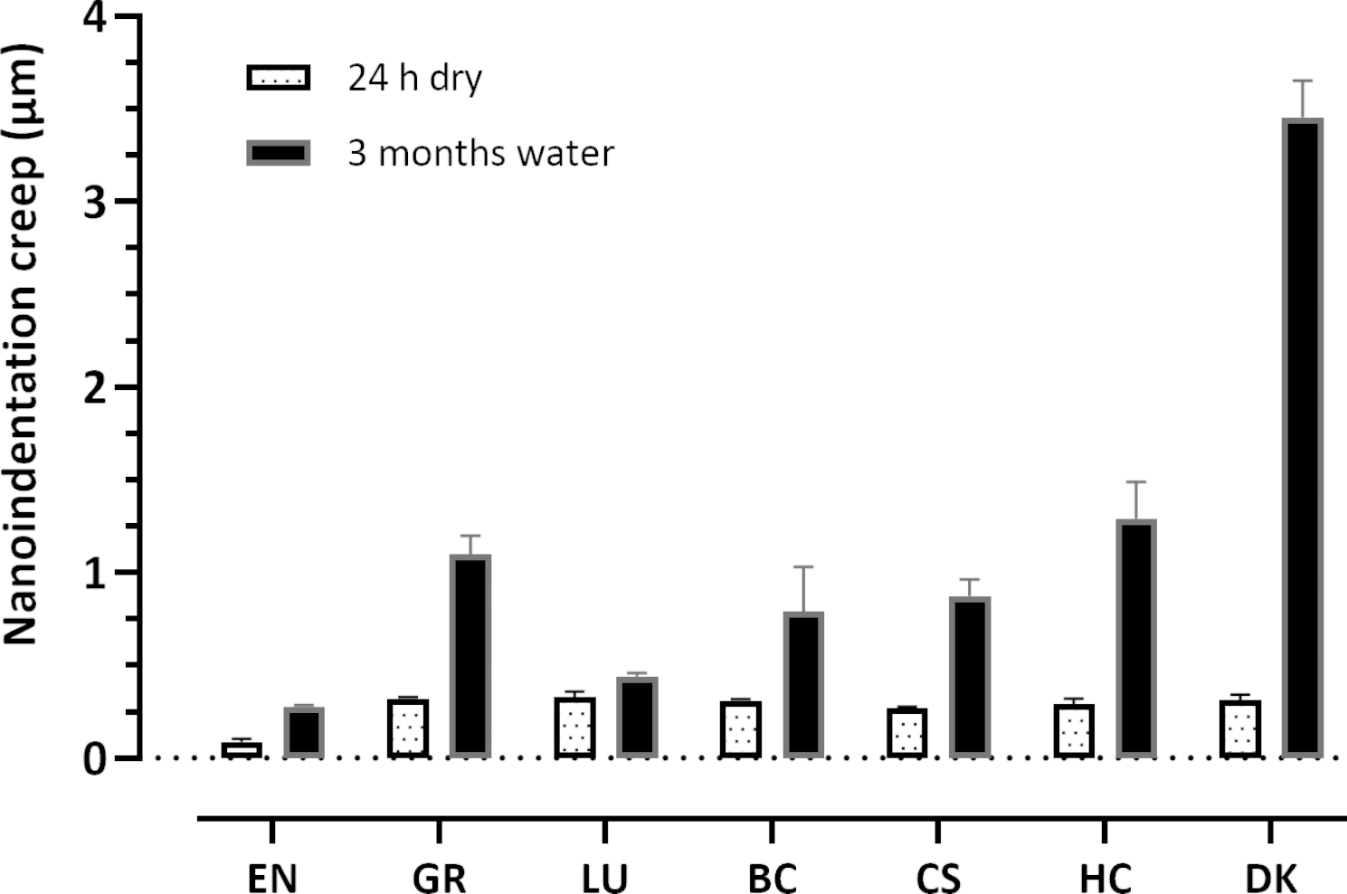
Nanoindentation creep (µm) of the two groups (24 h dry and 3 months water storage) of CAD-CAM composite blocks. Error bars represent the standard deviation

**Fig. 4:**
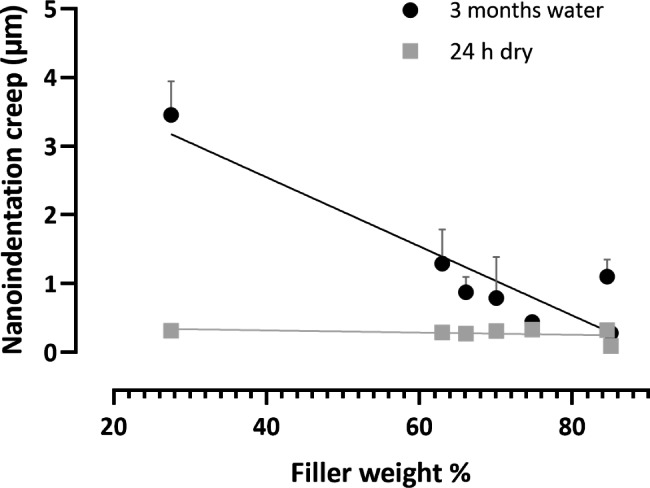
A scatter plot of nanoindentation creep (µm) and filler weight% (measured experimentally) for CAD-CAM composite blocks. There was a non-significant negative correlation between nanoindentation creep (µm) and filler weight% at 24 h dry storage (*P* = 0.2, R^2^ = 0.14) but a significant correlation at 3 months water storage (*P* = 0.005, R^2^ = 0.85 )

**Fig. 5:**
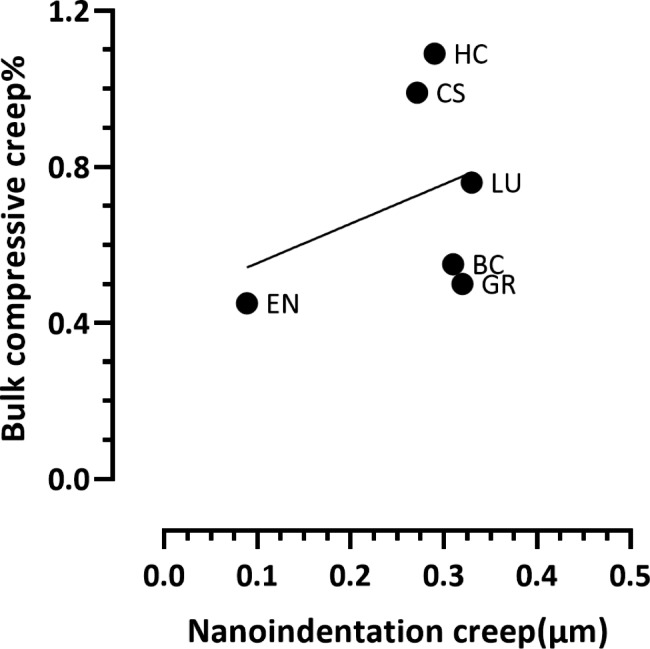
Nanoindentation and bulk compressive creep correlation for CAD-CAM composite blocks after 24 h dry storage, there was a non-significant (*P* = 0.51, R^2^ = 0.12) positive correlation between nanoindentation creep (µm) and bulk compressive creep (%)

**Fig. 6:**
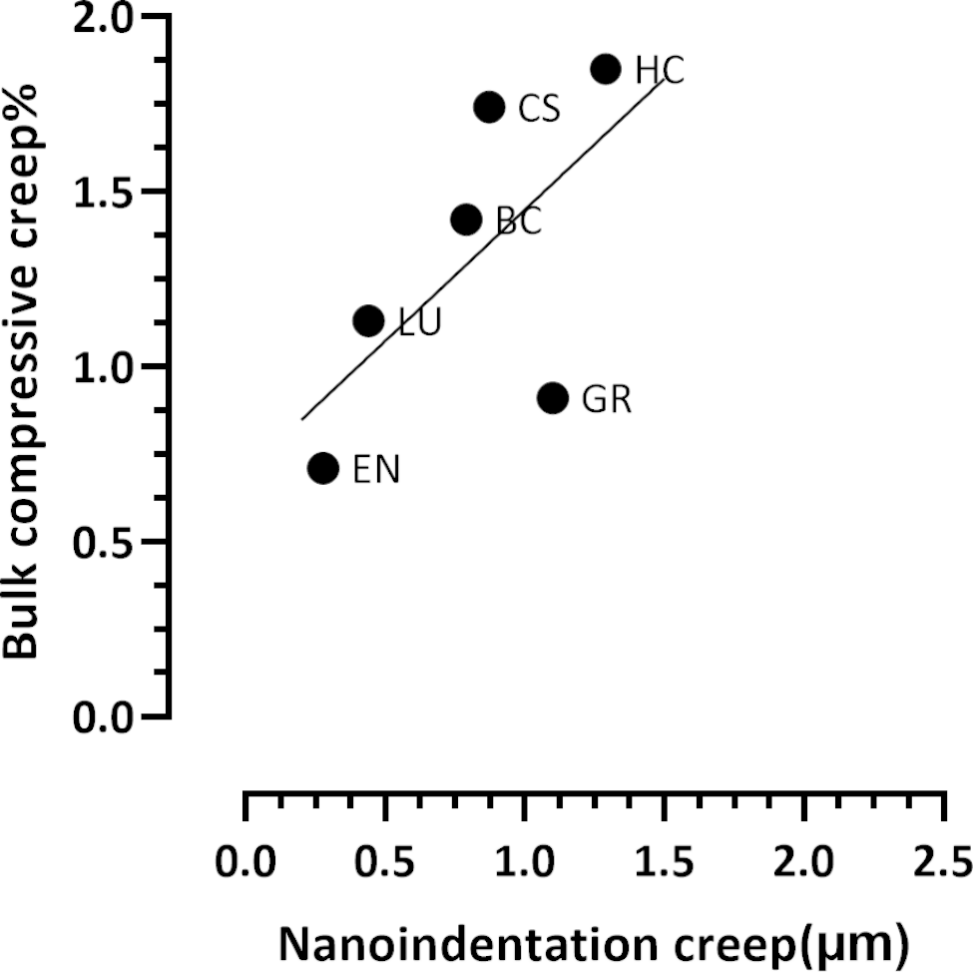
Nanoindentation and bulk compressive creep correlation for CAD-CAM composite blocks after 3 months water storage, there was a non-significant (*P* = 0.17, R^2^ = 0.40) positive correlation between nanoindentation creep (µm) and bulk compressive creep (%)

Two-way ANOVA results showed that each of the independent variables (CAD-CAM material and storage condition) or their interaction had a statistically significant effect (*P* < 0.001) on the measured nanoindentation. The greatest influence was for the CAD-CAM material (partial eta squared η_P_^2^ = 0.370) followed by the interaction effect (η_P_^2^ = 0.329) while storage condition had the lowest effect (η_P_^2^ = 0.289). The results are presented in Figs. 2, 3, 4, 5 and 6; Table 2. A typical force-displacement curve generated from the Nanovea nanoindenter at 24 h and after 3 months water storage is shown in Fig. 2.

**Table 2:** Nanoindentation creep (µm) of CAD-CAM composite blocks at 24 h dry storage at 23 ˚C and at three month water storage at 37 ˚C, compared to previously investigated bulk compressive creep [8], and nanohardness and elastic modulus at 24 h dry storage at 23 ˚C previously investigated [29]

Material *(Code)*		Nanoindentation creep in µm (SD)		Nanohardness in *GPa (SD)*	Elastic Modulus in *GPa (SD)*	Bulk compressive creep % (SD)	
		24 h dry	3 months water storage	24 h dry	24 h dry	24 h dry	3 months water storage
Polymer- infiltrated ceramic network (PICN)	Vita Enamic (EN)	0.09 (0.015)^A,1^	0.28 (0.009)^A,2^	3.1 (0.17)^A^	34.56 (1.4)^A^	0.45 (0.07)^A,1^	0.71 (0.1)^A,2^
**Resin-composite blocks (RCB)**	Grandio Blocs (GR)	0.32 (0.01)^B,1^	1.1 (0.1)^B, E, 2^	1.3 (0.08)^B^	14.8 (0.4)^B^	0.50 (0.12)^A,B,1^	0.91 (0.06)^A,2^
	Lava™ Ultimate (LU)	0.33 (0.03)^B,1^	0.44 (0.02)^A,D,2^	1.25 (0.05)^B^	12.14 (0.76)^B^	0.76 (0.08)^B,1^	1.13 (0.15)^A,2^
	BRILLIANT Crios (BC)	0.31 (0.009)^B,1^	0.79 (0.24)^B, D, ,2^	0.85 (0.008)^C^	10.98 (0.6)^C,D^	0.55 (0.06)^A,B 1^	1.42 (0.26)^B,C 2^
	Cerasmart (CS)	0.27 (0.006)^B,1^	0.87 (0.09)^B, D, F, 2^	0.81 (0.006)^C^	10.36 (0.17)^C,D,E^	0.99 (0.04)^C, D 1^	1.74 (0.19)^B,2^
	Block HC (HC)	0.29 (0.03)^B,1^	1.3 (0.2)^E, F, 2^	0.775 (0.031)^C^	8.79 (0.35)^C,D, E^	1.09 (0.09)^D,1^	1.85 (0.09)^B,2^
**Ceramic-filled PEEK**	Dentokeep (DK)	0.31 (0.03)^B,1^	3.46 (0.2)^C,2^	0.34 (0.03)^D^	3.43 (0.29)^D,E^	NA	NA

Values with the same superscript letters represent non-significant differences among different materials (Bonferroni post hoc tests (α = 0.05)). Values with the same numbers represent non-significant differences among the storage conditions for each material (Independent sample t-test)

Figure 3 shows the nanoindentation creep in µm after 24 h of dry storage and after three months of water storage for the investigated CAD-CAM blocks. The two-way ANOVA that examined the effect of storage and material type highlighted a statistically significant effect of storage and material type and significant interaction between the effects of storage and material (*P* < 0.0001).

The nanoindentation creep after 24 h storage ranged from 0.09 to 0.33 μm and increased after 3 m storage in distilled water to between 0.28 and 3.46 μm. There was a statistically significant difference in nanoindentation creep behaviour between the two storage media for each of the investigated materials (independent t test). (Fig. 3; Table 2).

At 24 h dry storage EN (which is a PICN) exhibited the lowest nanoindentation creep 0.09 (0.006) µm and showed a significant difference from all other investigated materials (RCBs). All RCBs showed comparable naoindentaytion creep in the following order LU > GR > DK = BC > HC > CS. After three months of water storage; EN still showed the lowest creep compared to all RCBs. LU showed the lowest creep of the resin based blocks followed by CS, BC, GR and HC respectively. DK with lowest filler weight% (27% w/w) had the highest creep 3.46 (0.2) µm after 3 months of water storage. There was a statistically significant difference between all the investigated materials after 3 months of water storage (Bonferroni post hoc). (Fig. 3; Table 2). There was a significant negative correlation between both nanoindentation creep (µm) and nanohardness at 24 h dry storage (*P* = 0.009, R^2^ = 0.77) and nanoindentation creep (µm) and nanohardness at 24 h dry storage (P = 0.006, R^2^ = 0.80).

There was a non-significant negative correlation between nanoindentation creep (µm) and filler weight% at 24 h dry storage (*P* = 0.2, R^2^ = 0.14) but a significant correlation at 3 months water storage (*P* = 0.005, R^2^ = 0.85) (Fig. 4).

There was a non-significant (*P* = 0.51, R^2^ = 0.12) positive correlation between nanoindentation creep (µm) and bulk compressive creep (%). Nevertheless, EN exhibited the lowest creep at the two; nano and macro scales. (Fig. 5). After three months of water storage there was a non-significant (*P* = 0.17, R^2^ = 0.40) positive correlation between nanoindentation creep (µm) and bulk compressive creep (%): EN exhibited the lowest creep and HC exhibited the highest creep at both nano and macro scales. (Fig. 6).

## Discussion

This study investigated five resin composite blocks, one polymer infiltrated ceramic network (PICN) and one ceramic-filled PEEK. Creep resistance indicates the viscoelastic stability of a material and its resistance to catastrophic failure under loading [33]. In this study the creep depth was measured initially at dry conditions to measure the nanoindentation creep depth before servicing in simulated oral conditions. Water was used at 37 ˚C, simulating intraoral fluids and temperature and being more reflective of clinical conditions [34]. According to ISO 10993-13 materials intended to be used for more than 30 days and tested in simulated oral conditions should be tested at 1 month, 3 months, 6 months, and 1 year [35], hence a short term storage of 3 months was used.

There was a statistically significant difference in the nanoindentation creep depth between the investigated materials. Thus, the first null hypothesis was thus rejected. The specimens stored for 3 months in 37 ± 1 ˚C distilled water showed an increased nanoindentation creep depth compared to those stored dry for 24 h at 23˚C. Two-way ANOVA showed a significant effect of water storage and material type (p < 0.0001). Hence, the second null hypothesis was rejected. Dental restorative materials are subjected to variable moisture levels, temperature and acidity levels in the oral cavity which could negatively influence longevity and clinical performance of them [34, 36]. Such intra oral changes might lead to degradation and reduction in material stiffness due to plasticization of the polymer matrix [37–39], subsequently jeopardising composite material mechanical and viscoelastic behaviour [40]. Storage in water can lead to water sorption thus increasing creep and reduced creep recovery of the composite materials [8].

The effect of filler loading on nanoindentation creep depth was investigated in this study. There was a non-significant negative correlation between nanoindentation creep (µm) and filler weight% at 24 h dry storage (P = 0.2, R^2^ = 0.14) but a significant correlation at 3 months water storage (*P* = 0.005, R^2^ = 0.85); thus, the third null hypothesis was rejected. It can be noted that the filler loading has more influential effect after 3 months storage than at dry condition except for PICN which showed lowest and significantly different nanonindentation creep after 24 h dry storage in agreement with similar studies [21, 41, 42]. The filler microstructure (volume percent, size and distribution) and resin matrix composition can influence the viscoelastic stability and mechanical properties of composite materials [43, 44]; PICN (EN) showed the lowest nanoindentation creep at both storage conditions (24 h dry and 3 months water storage) and this can be attributed to the three dimensional skeleton provided by filler and resin matrix infiltration as compared to other CAD-CAM composite blocks [45], and it reflects the material stability under loads especially in cases of bruxism where ceramics might fail due to brittleness [46]. There was a significant negative correlation between both nanoindentation creep (µm) and nanohardness (GPa) at 24 h dry storage (*P* = 0.009, R^2^ = 0.77) and nanoindentation creep (µm) and elastic modulus (GPa) at 24 h dry storage (P = 0.006, R^2^ = 0.80). EN exhibited higher hardeness and elastic modulus than RCBs and this trend was also noticed in nanonindenation creep. EN has similar hardness and elastic modulus to tooth structure and thus it can withstand elastic deformation and is more damage-tolerant [7, 17].

Compressive creep behaviour has been investigated previously, with PICN showing superior viscoelastic stability at wet and dry conditions compared to RCB [8]. At the nanoscale a study has investigated nanoindentation creep of experimental PICN and found it was comparable to human enamel [21]. Further, creep using Hertzian indentation of two experimental PICN materials with different filler loadings has been investigated [41]. In this study, DK (with the lowest filler weight%:27% w/w) has shown the highest creep after 3 months of water storage. As stated earlier the filler volume influences the material properties [43]. Nanoindentation creep of RCBs was ranked as the following: LU with filler content (74.8% w/w), HC (63% w/w), GR (84.6% w/w), CS (66% w/w) and BC (70% w/w). It was noted that nanoindentation creep was not linearly correlated to filler weight%, and this might be attributed to the indenter tip size relation to the filler particle size [47] and the applied load: in other words, with a very small indenter tip or inadequate load, the nanoindentation measurement might not reflect the bulk of material properties [27]. This can be overcome by using the appropriate indenter size along with appropriate load [48].

Nanoindentation and bulk compressive creep (data from a previous study) [8] for CAD-CAM composite blocks (except DK) showed a non-significant positive correlation [(*P* = 0.51, R^2^ = 0.12), (*P* = 0.17, R^2^ = 0.40)] after 24 h dry and 3 months water storage respectively and therefore the fourth null hypothesis was rejected. Comparing the two methods: ‘nanoindentation creep’ and ‘bulk compressive creep’ is feasible, however, the two methods are different in many aspects including the measurement scale: nanoindentation creep is considered a microscopic and localised measurement while bulk creep is macroscopic and might cause specimen deformation. The applied load, the force mode and magnitude all influence the creep degree (for instance, tensile load is more likely to cause fracture than compressive). Bulk compressive creep was undertaken constant stress (20 MPa for 2 h in the compared study), while for nanoindentation creep a load of 20 gf for 20 s was applied and the applied stress reduces as the indenter penetrates deeper into the specimen surface [49]. Also, the load magnitude and loading time could transform linear viscoelastic behaviour into nonlinear during moving from low to high magnitude load [50].

## Conclusions

The PICN material showed the highest dimensional stability in terms of nanoindentation creep depth in both storage conditions. On the other hand, RCBs and PEEK showed similar nanoindentation creep when dry, but demonstrated increased nanoindentation creep upon water storage. Filler loading has more influence on nanoindentation creep of CAD-CAM composite blocks upon water storage.

## Data Availability

The datasets used and/or analysed during the current study are available from the corresponding author on reasonable request.

## Abbreviations

CAD-CAM: Computer-aided design- Computer-aided manufacturing
PICN: Polymer-infiltrated ceramic network
RCB: Resin composite block
PEEK: Polyetheretherketone
ANOVA: Analysis of variance
EN: Vita Enamic
GR: Grandio Blocs
LU: Lava Ultimate
BC: Brilliant Crios
CS: Cerasmart
HC: Block HC
DK: Dentokeep
